# Functional Magnetic Resonance Imaging in the Olfactory Perception of the Same Stimuli

**DOI:** 10.3390/life11010011

**Published:** 2020-12-25

**Authors:** Andrea Ciorba, Stavros Hatzopoulos, Cristina Cogliandolo, Chiara Bianchini, Martina Renna, Luca Perrucci, Magdalena Skarzynska, Piotr Henryk Skarżyński, Paolo Campioni, Corrado Cittanti, Aldo Carnevale, Melchiore Giganti, Stefano Pelucchi

**Affiliations:** 1 ENT and Audiology Unit, Department of Neuroscience and Rehabilitation, University Hospital of Ferrara, Via Aldo Moro, 8, 44124 Cona FE, Italy; sdh1@unife.it (S.H.); cristinacogliandolo@gmail.com (C.C.); chiara.bianchini@unife.it (C.B.); martinarnn@gmail.com (M.R.); stefano.pelucchi@unife.it (S.P.); 2 Radiology Unit, Department of Traslational Medicine, University Hospital of Ferrara, Via Aldo Moro, 8, 44124 Cona FE, Italy; luca.perrucci@edu.unife.it (L.P.); paolo.campioni@unife.it (P.C.); corrado.cittanti@unife.it (C.C.); aldo.carnevale@unife.it (A.C.); melchiorre.giganti@unife.it (M.G.); 3 Institute of Physiology and Pathology of Hearing, Maurycego Mochnackiego 10, 02-042 Warszawa, Poland; m.skarzynska@csim.pl (M.S.); p.skarzynski@ifps.org.pl (P.H.S.); 4 Institute of Sensory Organs, 05-830 Kajetany, Poland; 5 Department of Heart Failure and Cardiac Rehabilitation, Medical University of Warsaw, Żwirki i Wigury 61, 02-091 Warszawa, Poland

**Keywords:** olfaction, functional magnetic resonance imaging, fMRI, left anterior insula

## Abstract

Background. Data in the literature report that a number of studies have attempted to identify the exact location of the cortical olfaction representation, searching for evidence suggesting that sniffing odors can initiate a primary activation of the piriform cortex and the insula. Nowadays, due to the SARS-CoV-2 (COVID-19) outbreak, the functional study of the olfactory system could offer a better understanding of the physiopathology of olfactory perception, elucidating better the possible site(s) of damage induced by the COVID-19 infection. The aim of this paper was to evaluate brain maps generated from functional Magnetic Resonance Imaging (fMRI) data, collected from healthy individuals in response to the same olfactory stimulus. Methods. A total of 45 healthy volunteers, without history and/or no clinical signs of sinonasal disease and without history and/or presence of olfactory dysfunction underwent fMRI assessment. Subjects were presented with the same odorous stimuli at specific intervals. fMRI generated brain maps were used in the identification of different cortical areas, involved in the stimuli perception. Results. The fMRI brain maps showed that odorous stimuli activate primarily the left anterior insula (in 35/45 cases or 77.8%). Other activated areas include: the low temporal gyri, the middle and superior temporal gyri, the frontal and piriform cortex, the anterior cingulate gyrus, the parahippocampal gyrus, the temporopolar area, the para-insular area, the subcentral area, the supramarginal gyrus, the occipital cortex and the cerebellum. Conclusions. fMRI resulted as a safe and reliable means to study the perception of olfaction in the cortex. The data of this study suggest that the anterior insula is the main stimulated area when olfactory stimuli are present. This area is always activated, despite the hand and nostril dominance.

## 1. Introduction

The olfactory system has been studied with functional Magnetic Resonance Imaging (fMRI) using different protocol approaches [1,2,3].

Data from the majority of the fMRI studies report that after a period of 10–15 s post-stimulus, an activation of the primary olfactory cortex is visible, after which the Blood Oxygenation Level Dependent signal (BOLD) decreases to a baseline level. Clinical history, habituation and sinonasal diseases contribute not only to an altered perception of olfaction but to a weak piriform cortex activation, observed in many fMRI measurements. The most common procedure of data analysis in functional brain imaging, is called “general linear model” (GLM). The latter is used to generate brain maps, indicating those areas statistically correlated with the stimulus paradigm. The Region of Interest analysis (ROI), permits a more reliable evaluation of the activated brain regions [1,2,3]. The Activation Likelihood Estimation (ALE) method, statistically merges data from neuroimaging studies and reports a comprehensive probability map [1,2,3]. Functional magnetic resonance imaging (fMRI) reveals human brain areas, which are activated after a specific task or stimulus, using blood as a natural contrast. Neuronal activity results in a higher consumption of oxygen and glucose in the blood. The deoxy-hemoglobin (dHB) is paramagnetic, producing a short T2*relaxation time, whereas the oxyhemoglobin (O_2_Hb) has a diamagnetic behavior. A rapid series of scans analyze the dynamic variation of blood flow using the oxy/deoxy-Hb ratio, via fast echo planar imaging pulse sequences (EPI), detecting the blood oxygenation level signal (BOLD) and consequently the brain activity [4,5]. A stimulus protocol/paradigm is a temporal succession of stimuli, evoking in the tested subject a specific cortical response, during the fMRI assessment. The stimulus paradigms can be of two types: (i) a block protocol design where sequences, with the stimulus present or not present, are alternated throughout the assessment; (ii) an event-related protocol design, where during the assessment the stimulus is randomly present [4,5]. 

Nonetheless, the human olfaction neurobiology still needs to be additionally detailed; even if neuroimaging studies have identified several structures involved, the available data report numerous discrepancies primarily on the brain areas which are specifically activated by olfactory stimuli. Until now, a detailed functional organization has not been identified [6,7,8]. The anterior insular cortex is reported to play a major role in the sensory processing of olfaction; the anterior piriform cortex should reflect the chemosensory composition, while the posterior piriform cortex should compare odor features [4,5,6,7]. Other reported areas are the amygdala, which could be involved in the emotional processing of olfactory stimuli and the posterior orbitofrontal and anteromedial temporal lobes, which have been reported to be involved in odor identification, discrimination, and memory [6,7,8].

The objective of this study was to evaluate fMRI-generated brain maps, from healthy individuals, stimulated by the same olfactory stimulus. 

## 2. Subjects and Methods

### 2.1. Study Design and Subjects

Prospective study, designed to evoke an activation of olfactory brain areas. A total of 45 young, healthy volunteers were enrolled. Each subject underwent: (i) an assessment of medical history in order to exclude previous instances of olfactory disorders and/or presence of olfactory disorders/dysfunctions; (ii) an ENT evaluation with nasal endoscopy, in order to exclude current sinonasal pathologies. All 45 subjects passed the clinical inclusion criteria and underwent fMRI assessment. 

The testing procedures were in accordance with the ethical standards of our institutional Committee and with the 1964 Helsinki Declaration and its later amendments or comparable ethical standards. The study was approved by the Ethics Committee of the University of Ferrara (reference number 171183). Furthermore, each subject compiled a written consent form in order to participate in the testing.

### 2.2. Stimulus 

The stimulus was a “coffee odor” placed via a cotton rod approximately at a distance of 1 cm from the nasal tip. All subjects were instructed to keep the cotton rod in their right hand without smelling it and to put it close to the nose tip, when they were asked. An operator instructed each subject to breathe normally (i.e., without any sniffing) and to focus on the perception of the odor. In another room, the standardized coffee essence was prepared by leaving the cotton rod immersed in a coffee jug for 1 min and then placed outside for 5 min. Then, a second operator gave the rod to the subject, who was already prepared for the fMRI scan. The rationale for this procedure was to prevent the tested subjects from smelling the coffee odor prior to stimulation.

### 2.3. Imaging Protocol

All subjects were informed not to drink, eat or smoke for 3 h prior to testing. All fMRI scans were obtained using a 1,5 Tesla Signa HDX (General Electric Healthcare, Milwaukee, WI, USA) and an 8-channel head-coil. The scanning protocol consisted in a morphological SPoiledGRadient-echo 3D T1-weighted sequence (matrix size = 256 × 256; slice thickness = 1.2 mm; NEX = 1; flip angle = 13°) and a functional acquisition sequence EcoPlanarImaging-GradientEcho (BOLD–multiplanar, matrix size = 64 × 64, slice thickness = 5 mm, slice gap = 1 mm, NEX = 1; Repetition time (TR) = 3000, Echo time (TE) = 60 ms, FA = 90°). Slices were oriented along the Anterior Commissure–Posterior commissure line. The first sequence searched for any macroscopic alteration and overlapping with the functional map, for a better characterization of activated cortical areas. 

### 2.4. Investigation

**Figure 1:**
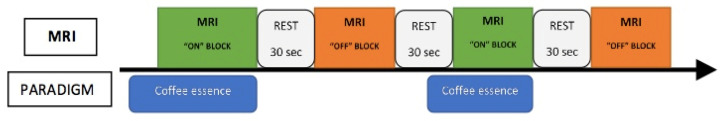
Experimental design. The complete functional MRI scanning period took 2 min and 58 s. A single whole brain scan lasted approximately 3 s. The first block consisted of 8 whole brain scans and started ten seconds after smelling the coffee odor. A 30 s rest pause followed, while the subject kept the rod along the body and the olfactory area reached the baseline level. The second block was with no stimulus and consisted in 5 whole brain scans. In a second rest-break, the subject was invited to place the rod again close to the nasal tip. A third “on” block was composed by 5 scans. The last acquisition, successive to a rest-break of 30 s with no stimulus, consisted in 5 scans. Subjects were recommended to breathe regularly throughout the examination and be motionless, excepting during the rest phases when they were asked to move the rod.

During testing, subjects were laid in a supine position with their eyes closed and were asked to remain motionless and if possible, not to swallow. The fMRI block stimulus paradigm [8] consisted of 4 blocks with a 16–20 s duration, having an inter-stimulus interval of 30 s. The first block, consisted of 8 whole brain scans and the following three blocks of 5 scans, for a total of 23 scans per stimulus. The fMRI scanning assessment lasted approximately 178 s (2 min and 58 s). A single whole-brain scan lasted approximately 3 s. The acquisition sequence was initiated after smelling the rod for approximately ten seconds. A 30 s rest pause followed, while the subject kept the rod along the body to allow the olfactory areas to reach the baseline levels. Therefore, the second block was an “off” stimulus scans. A second rest break was performed to put again the rod close to the nose before getting the third “on” stimulus block. The last acquisition followed a rest pause of 30 s with no stimulus. At the end of testing, subjects were asked about the type of odor experience and about the predominant (left or right nostril) side (Figure 1).

### 2.5. Post-Processing

**Figure 2:**
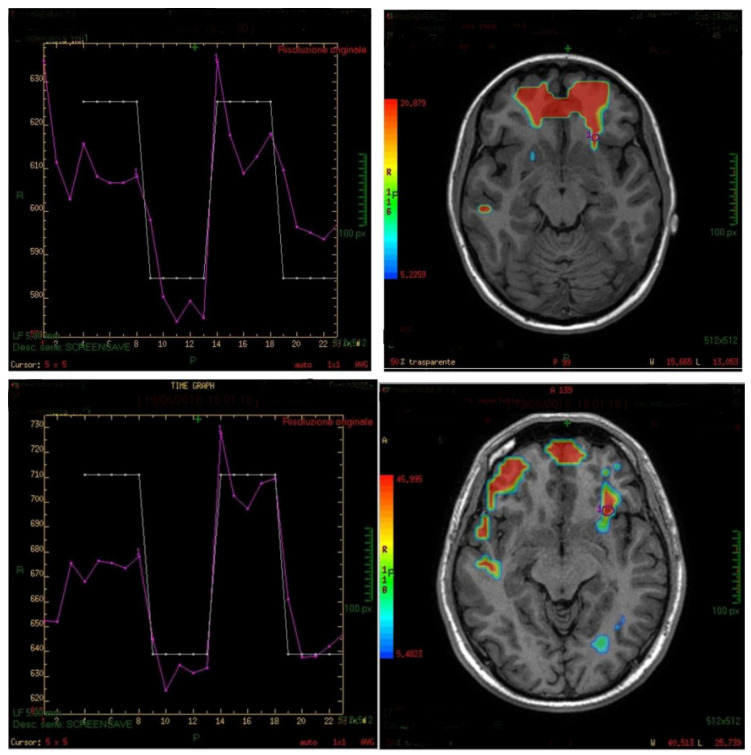
Examples of region of interest (ROI) analyses of the left insula (violet lines in left plots) linked to the protocol paradigm (white lines) in two patients. The maps indicate that the areas of the left insula and some other nearby regions have been activated during the experiment. In the right upper corner, the map shows the subgenual area activation, whereas in the map below, an activation of the right hemisphere from the forehead to the back is visible, including the area 45 (BA45) and the superior temporal gyri (STG) and middle temporal gyri (MTG).

A GE Advance Windows workstation with the Functool software was used for image processing and for the elaboration of the fMRI brain maps. The first 3 brain scans were discarded in order to eliminate any initial transit-signal fluctuations. Active areas on the fMRI map were defined using the sectional Atlas and Brain Tutor 3D. A region of interest (ROI), which was set on the anterior insula and background, allowed the verification of possible artifacts in the BOLD signal such as drift and spikes (Figure 2).

### 2.6. Statistical Analysis

An Excel spreadsheet was used for data input. The data were analyzed using SPSS 24 (Windows Base System). Associations between variables were calculated using the Chi-Square test. Statistical significance was considered at *p* < 0.05.

## 3. Results

Participants were 26 women and 19 men (average 27.63 years; age range 22 to 48 years); 5 were left-handed and 40 right-handed. None of the participants declared any history or presence of olfactory disorders. The ENT clinical evaluation of the participants showed the following: septum scoliosis in 73.3% (66.7% right and the 33.3% left) and a concha bullosa in 4.4%; turbinate hypertrophy in 48.8% of subjects; 13.3% presented a minor mucosal sinus hypertrophy and only 2 subjects presented an adenoid hypertrophy without nasopharyngeal obstruction.

All subjects recognized the coffee odor, and 84.4% referred to have a dominant nostril (right nostril in 63.1% of cases and the left in the remaining 36.9%). 

During the fMRI evaluation, only one patient reported an unpleasant sensation (2.2%), and another one reported dysgeusia. 

**Figure 3:**
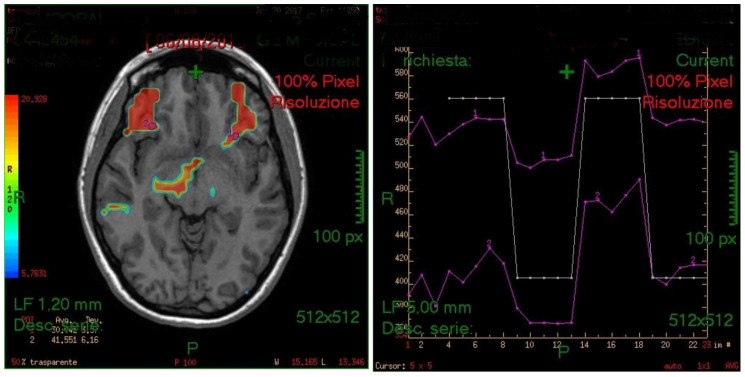
Map showing a bilateral activation of the anterior insula in a right-handed subject.

**Figure 4:**
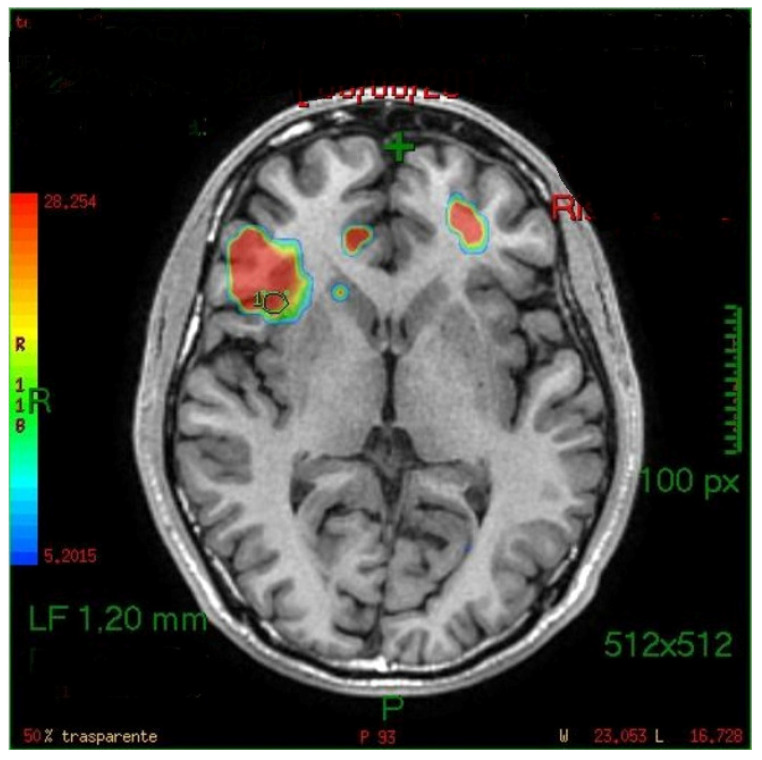
Right insula activation in a left-handed subject.

The generated fMRI brain maps suggested that the main activated area, by the stimulus, was the anterior insula (in 35/45 cases or 77.8%) Other activated areas included: the inferior temporal gyri (ITG), the middle (MTG) and superior temporal gyri (STG), the frontal and piriform cortex, the anterior cingulate gyrus, the parahippocampal gyrus, the temporopolar area, the parainsular area, the subcentral area, the supramarginal gyrus, the occipital cortex and the cerebellum (see also Figure 2, Figure 3 and Figure 4). In the majority of the subjects, the olfactory area which was most consistently activated was the left side insula. The activation of the left hemisphere was present in 81.5% of the subjects (*p* < 0.005).

There was no significant association between hand dominance and nostril dominance (*p* = 0.138). Possible associations between other activated areas and the dominant hand were evaluated, however, all results were not significant (see also Table 1). The relationship between the other activated areas and the dominant nostril resulted also as nonsignificant (see Table 2).

## 4. Discussion

Due to the SARS-CoV-2 (COVID-19) outbreak, the functional study of the olfactory system is ‘back in vogue’ nowadays, since anosmia and dysosmia are common reported symptoms of the infection and can persist for months. Only a better comprehension of the physiopathology of olfaction can offer the possibility to elucidate in detail the probable site(s) of damage induced by the COVID-19 infection. 

Even if studies on the exact location of the cortical olfaction-representation are dated back to 80 s and 90 s, the human olfaction neurobiology still needs to be updated with additional details. Levy et al. (1997) attempted to measure quantitatively the cerebral activation during an olfactory stimulus, studying possible differences among men and women. They recorded an activation of other areas such as frontal cortex, cingulate cortex and other components of limbic system, possibly due to the presence of integrative emotional somatic-sensitive components [9]. Vedaei et al. (2013) analyzed the olfaction of a group of studies by fMRI [3]. They focused on the different possibility of smell perception: “sniffing” or “smelling” and noticed two distinct patterns of cerebral activation. Sniffing induced the activation of the piriform cortex and the orbito-frontal cortex, even at the absence of odorous stimuli [10]. The smelling perception involved different cerebral areas, such as the piriform cortex, the amygdala, the insula orbito-frontal cortex, the cingulate cortex and the right thalamus. Factors that can influence the cerebral response to an olfactory stimulus are the habituation or memory. For example, data in the literature show that the BOLD (Blood oxygenation level dependent) signal is reduced due to habituation, and that the activation of the piriform cortex could be inconsistent due to fMRI artifacts and stimulus sequence parameters [11]. 

It was reported that cortical activation could involve memory patterns; therefore, other areas such as the right hippocampus, the inferior frontal right lap and the fusiform middle lap could be involved in the olfaction experience [12]. Regarding sensations such as the pleasure or discomfort related to different odors, it has been reported that areas involved in cases of good odors, like the orbito-frontal cortex are not activated in cases of unpleasant ones [13]. 

Our data are consistent with other recent neuroimaging studies of olfaction, relating sniffing odors with a primary activation of the piriform cortex and the insula [14]. In particular, the data of the present study showed that the left anterior insula is always the main stimulated olfactory area, when presenting the stimuli; this area is mainly activated, despite the hand dominance and the nostril dominance.

Concerning our protocol, it was adapted from the experience of previous studies, which carefully evaluated the image acquisition of the fMRI technique; images were acquired at 10 s after the initiation of the olfactory stimulation, as in previous experimental setups by Poellinger et al. (time set = 9 s), Lambion (time set = 9 s) and Savic (time set = 15 s) [11].

A possible drawback of this study is related to the fact that there was a technical limit of compatibility between the DICOM of the MRI machine and the external software (SPM-MATLAB Portal); therefore, it was decided to use a dedicated Workstation of the same manufacturer (RM of GE) for the maps, which were accurately evaluated.

Understanding olfaction pathophysiology, including its neuromodulation, is crucial particularly in defining olfaction diseases, which are more frequent and impactful than described. Two types of olfaction diseases are mainly described, qualitative and quantitative defects, with the latter more frequent, especially in older people, affecting from 3% to 20% of population [15,16]. A reduction or an impairment of the olfaction is reported to have a severe impact on quality of life [17]; olfaction provides different information, from the taste perception of food and drinks [18] to protection from dangers as burnings or gas leaks [19,20] and it is also fundamental for some professional figures like cooks, wine tasters, perfumers, nurses or firemen [17]. Olfactory dysfunction has been also related to scarce personal hygiene [21,22], impaired sex life [23], and limited emotional feelings [18]. Furthermore, the functional impairment of the cortical areas involved in the olfaction has also been related to several conditions. Ageing itself has been related to an important decline of primary olfactory cortex activity, by Cerf-Ducastel et al. [24]. Potentially, fMRI could be also used in order to evaluate the functional severity of a cerebral disease. In particular, several fMRI studies have reported the presence of an olfactory decline in subjects with Dementia and Alzheimer Disease; Wang and colleagues showed that olfaction declines progressively with the disease [25]. Also, subjects affected by Parkinson’s disease have significant decline of the primary olfactory cortex activity, and particularly in the amygdala and in the hippocampus, both with the presence of a pleasant or unpleasant olfactory stimulus. This finding can be due to a lower sensibility of those regions to emotional stimuli in cases of PD [26]. On the other hand schizophrenic individuals retain the highest levels of olfactory processes like frontal cortex, temporal cortex and cingulate cortex, according to Schneider et al. [26]. 

Another possible clinical application of fMRI is olfactory training, in order to evaluate the neural pathways (re)activated during the olfactory perception. This is a mix of exercises to do in order to empower olfaction in case of its impairment. To test this technique, Kollndorfer et al., used the fMRI in order to evidence the networks activation during the olfactory perception in a group of healthy controls, comparing it with anosmic patients, before and after olfactory training. The results were that after the training period the number of connections to the seed region of the olfaction network increased, and some functional connections in the olfactory network were re-established. Those findings allowed authors to declare that olfactory training may increase smell performance, having effects in neuronal plasticity [27].

## 5. Conclusions

The data of this study suggest that: (i) fMRI is a safe and reliable means to study the cortical olfaction stimulation; (ii) the left anterior insula is the main stimulated olfactory area when presenting the stimuli. This area is always activated, despite the hand and the nostril dominance.

More detailed data about the cortical olfaction perception could also be helpful in (i) determining the validity and nature of olfactory area diseases, (ii) monitoring changes of the olfactory function over time (including influences of pharmacological, surgical, or immunological interventions), (iii) detecting olfactory dysfunction and compensation (iv) investigate anosmic-hyposmic patients.

## Figures and Tables

**Table 1:** Possible associations between activated areas and the dominant hand.

Associations	Significance
Area 22/dominant hand	*p* = 0.231
Area 21/dominant hand	*p* = 0.770
Area 20/dominant hand	*p* = 0.520

**Table 2:** Possible associations between activated areas and the dominant nostril.

Associations	Significance
Area 22/dominant nostril	*p* = 0.524
Area 21/dominant nostril	*p* = 0.326
Area 20/dominant nostril	*p* = 0.231

## Data Availability

The data presented in this study are available on request from the corresponding author. The data are not publicly available due to privacy reasons.
